# Psychosocial assessment and follow-up in vascularized composite allotransplantation candidates: clinical observations from the Finnish experience

**DOI:** 10.3389/frtra.2026.1845139

**Published:** 2026-09-16

**Authors:** Benita Antas, Jan Nyberg

**Affiliations:** Department of Psychiatry, Helsinki University Hospital, Helsinki, Finland

**Keywords:** longitudinal, mentalization, psychosocial, transplantation, VCA

## Abstract

Psychosocial assessment is increasingly recognized as a central component in the evaluation of candidates for vascularized composite allotransplantation (VCA), yet published clinical perspectives remain limited. Drawing on longitudinal clinical observations of five candidates assessed for VCA, three of whom subsequently underwent transplantation in the Finnish VCA program at Helsinki University Hospital, this article reflects on key considerations shaping psychosocial assessment and follow-up. Psychosocial assessment in VCA requires longitudinal, multidisciplinary information gathering, with the clinical picture evolving across successive encounters. Three interrelated domains appear particularly relevant to clinical assessment: psychosocial functioning, cognitive capacity, and social context. We further propose that the concept of mentalization may offer a useful lens for understanding individuals’ abilities to reflect on their illness, expectations, interpersonal relationships, and possible changes following transplantation.

## 1 Introduction

Vascularized composite allotransplantation (VCA), including face and hand transplantation, represents one of the most complex and ethically nuanced areas of reconstructive surgery. Unlike life-saving solid organ transplantation, VCA is primarily life-enhancing, aiming to restore function, appearance, and social participation for individuals with severe disfigurement or limb loss. This distinction has important psychosocial implications: recipients are exposed to lifelong immunosuppression and its associated risks in exchange for benefits that are functional, relational, and existential rather than survival-based (1–6).

Psychosocial assessment is central to clinical decision-making in VCA programs. International consensus recommendations emphasize standardized, multidisciplinary approaches to candidate selection, assessment, follow-up, and outcome evaluation in face transplantation (7). Although current frameworks highlight psychiatric stability, adherence potential, social support, and realistic expectations, they may not fully capture the dynamic psychological processes involved in VCA (1–4, 6).

VCA procedures in Finland are centralized at Helsinki University Hospital (HUS). Finland's publicly funded healthcare system provides a stable framework for long-term follow-up, potentially reducing barriers described elsewhere (5). Supportive attitudes toward organ donation and media emphasis on patient dignity also shape the Finnish VCA context.

In this article, we describe how the psychosocial assessment and follow-up process unfolds at Helsinki University Hospital (HUS), highlight clinical observations emerging from this experience, and propose core domains and content areas that may be relevant for psychosocial assessment in VCA candidates.

## 2 Article approach and clinical basis

This perspective draws on clinical work with five candidates evaluated within the Finnish VCA program at HUS since 2013, three of whom subsequently underwent transplantation. The observations are based on clinical interviews, structured psychiatric assessments, standardized psychological measures, one neuropsychological evaluation, and longitudinal follow-up, with the clinical observations primarily concerning transplant recipients because of their longer and more intensive follow-up.

This article is not based on a research study. Rather, it presents observations derived from routine clinical care and professional reflection on the psychosocial assessment and follow-up process. All patients provided written informed consent for the use of their information for educational and scientific purposes. All data have been anonymized. To protect privacy, diagnostic and medication-related information is reported only in general terms.

During the evaluation phase, all candidates underwent structured psychiatric interviews using the Structured Clinical Interview for DSM Disorders (SCID-I and SCID-II) and completed several standardized self-report measures as part of the psychological assessment, including: the Impact of Event Scale (IES), the Resilience Scale (RS), the Body Appreciation Scale (BAS), the Coping Orientation to Problems Experienced Inventory (COPE), and the Difficulties in Emotion Regulation Scale (DERS). For some candidates, the assessment was further supplemented with the Personality Assessment Inventory (PAI) and the Wartegg Drawing Completion Test. Follow-up assessments have relied primarily on longitudinal clinical observations rather than repeated structured measures. Health-related quality of life was assessed with the 15D instrument and monitored longitudinally among transplant recipients. No structured capacity assessment tool was employed.

## 3 Multidisciplinary psychosocial assessment process in the Finnish VCA program

There is no universally agreed set of psychosocial domains or assessment instruments for VCA candidate evaluation. Previous work in hand transplantation has described assessment of, among other domains, personality, cognitive functioning, mood, behavioral adherence, social support, and substance use (8). The multidisciplinary process integrates medical and psychiatric history with assessment of psychosocial functioning, understanding of transplantation, social support, and broader life circumstances.

### 3.1 Appraisal of psychosocial factors by the surgical team

The surgical team conducts the initial appraisal, and candidates considered potentially suitable are referred for further psychological and psychiatric assessment.

Candidates considered potentially suitable by the surgical team typically showed stable premorbid functioning, including prior engagement in work, preserved interpersonal relationships, and the ability to maintain social networks following orofacial dysfunction and disfigurement or limb loss. Adaptive psychological characteristics included perseverance and psychological flexibility, reflected in the ability to maintain perspective, tolerate uncertainty, and sustain a sense of humor. Culturally embedded constructs such as *sisu* may manifest clinically as persistent determination in the face of adversity. Motivation and stamina for extensive surgery and long-term rehabilitation, as well as adequate cognitive capacity, were deemed essential.

Maladaptive patterns—such as chronic bitterness, externalization of blame, fixation on minor grievances, or rigid, problem-saturated thinking—were deemed unfavorable. Severe depressive symptoms, hopelessness, or unrealistic expectations (e.g., viewing transplantation as a comprehensive solution to broader life difficulties) raised additional concerns. Significant substance misuse has been considered a contraindication.

These factors were assessed iteratively in several appointments. Because VCA is life-enhancing rather than life-saving, candidates should have substantial quality-of-life impairment while retaining sufficient functioning and adaptive capacity to engage in treatment and rehabilitation. Prior literature suggests that effective coping and realistic expectations are favorable (1–4, 6). Based on clinical experience, persistent functional and psychosocial challenges may provide an additional impetus to engage in treatment.

The surgical team also monitors psychosocial adaptation, quality of life, and engagement during rehabilitation and follow-up.

The perspective summarized in this section reflects clinical appraisals by plastic surgery chief physician Patrik Lassus and hand surgeon Jorma Ryhänen (personal communication, 2026).

### 3.2 Preoperative psychological assessment

The psychological assessment was conducted by the first author, a clinical psychologist, and included a semi-structured interview and standardized psychological measures. The interview addressed psychological difficulties, trauma, personality traits, impulse control, and emotional regulation, with particular attention to body image.

Attention was also paid to mentalization, emotional awareness, assertiveness, and humor because of their potential relevance to candidates’ realistic expectations, coping with the prolonged and demanding transplantation process, sustained treatment engagement, and capacity to communicate their needs and symptoms during rehabilitation.

Mentalization refers to the capacity to understand one's own and others’ behavior in terms of underlying mental states (9). It was explored through candidates’ reflections on emotions, intentions, expectations, and interpersonal perspectives during the interview. Difficulties in identifying and describing emotions were considered potentially relevant, as alexithymia has been associated with lower reflective functioning and impaired mentalization (10, 11). Assertiveness was considered in relation to active participation in healthcare and communication of needs and concerns, consistent with the concept of patient self-advocacy (12, 13). Use of humor was considered as a potentially relevant coping-related characteristic, while recognizing that its adaptive value depends on its form and context (14).

Cognitive capacity is assessed to ensure that candidates can understand and apply complex information throughout the transplantation process. Although only one candidate underwent formal neuropsychological testing, cognitive functioning was assessed using, for example, anamnestic information, including educational and occupational history, and clinical observations of the candidate's comprehension, retention of information, consistency across interviews, and ability to remember appointments. Suspected brain injury or organic disease warrants particular attention because it may impair understanding and complex self-care.

As part of the current assessment process, candidates are encouraged to express their psychological support needs and preferences for both the pre-and post-transplant phases. The assessment of the candidate's social context addresses the availability and quality of support, the social network's attitudes toward transplantation and its risks, relational stressors, and experiences of peer and professional support.

### 3.3 Preoperative psychiatric assessment

In the candidates evaluated, the psychiatric assessment addressed past and current mental disorders, symptom severity, substance use, social support, understanding and expectations of transplantation, and potential adherence concerns. The assessments were conducted by a psychiatrist specialized in consultation-liaison psychiatry, using both structured and semi-structured interviews.

Psychiatric disorders in themselves do not constitute an absolute contraindication for proceeding with VCA. However, the transplantation procedure, postoperative hospitalization—including the risk of delirium—and the demands of intensive rehabilitation place considerable psychological strain on recipients and require substantial resilience and adaptability. Psychiatric conditions may compromise adherence to the prolonged medical and rehabilitative regimens (15), while perioperative stressors and treatment setbacks may exacerbate symptoms, including suicidality, in vulnerable individuals. Thorough evaluation of both historical and current symptom severity, as well as the impact of prior and ongoing psychiatric treatment and rehabilitation, is therefore essential.

Assessment of candidates’ understanding and expectations may be supported by the teach-back method, in which individuals are asked to describe the procedure, associated risks, and follow-up requirements in their own words. Evaluation of decision-making capacity typically considers four abilities: understanding relevant information, appreciating its implications for one's own situation, reasoning about the available options, and communicating a choice (16). Severe psychiatric disorders, impaired impulse control, or significant cognitive deficits may compromise decision-making capacity in the VCA context.

Because sustained adherence—particularly to immunosuppressive medication—is a key predictor of post-transplant outcomes, prior treatment adherence should be examined carefully through interview and review of medical records, as poor pre-transplant adherence is associated with difficulties afterward (17, 18).

## 4 Clinical observations across the VCA process

Candidates assessed and monitored within the Finnish VCA program have been followed for varying durations, with the longest combined assessment and follow-up period exceeding six years. Three candidates have undergone either face or hand transplantation. One candidate withdrew because the anticipated surgery and its potential complications, including graft loss, were considered to risk an outcome worse than the preoperative condition. Another completed the psychosocial assessment and is awaiting transplantation.

### 4.1 Psychiatric morbidity among VCA candidates

Psychiatric symptoms observed during the assessment period were limited to depressive and adjustment-related symptoms and, when present, were mild in severity.

No trauma-related disorders have been identified. Although a history of suicidality was identified within the candidate group, no active suicidality was observed at the time of evaluation or during follow-up. Psychotropic medication use has been modest. Psychological support has generally involved intermittent, low-frequency outpatient follow-up, while postoperative inpatient care has included more frequent contact, up to two meetings per week.

### 4.2 Preoperative and early clinical observations regarding expectations

Candidates referred for psychosocial assessment have been carefully selected by the surgical team, and expectations regarding transplantation have generally been realistic. Candidates’ primary goals have emphasized functional recovery over appearance, including, for example, the ability to breathe through the nose following facial transplantation or increased independence in daily activities after hand transplantation. Psychological distress directly related to tissue loss has often been processed prior to transplantation, and the loss itself has in many cases been meaningfully acknowledged and grieved. Concerns related to aesthetic outcomes have typically been secondary, although individual experiences vary. Two recipients expressed satisfaction with specific aspects of the graft's appearance.

### 4.3 Resilience, coping, and interpersonal functioning

Recipients have demonstrated high levels of resilience and sustained motivation throughout evaluation, surgery, and rehabilitation. Use of humor has emerged as an important emotional resource. Levels of assertiveness varied. Appropriate assertiveness appears to be a beneficial characteristic in navigating the transplantation process. Some variation has also been observed in mentalization capacity. When needed, candidates have been supported in exploring and preparing for life after transplantation, including anticipated changes in body image, functional abilities, social interactions, and rehabilitation demands.

### 4.4 Social context and support needs

Close relatives and friends were considered important, while the need for professional psychological support varied. Some individuals have engaged in long-term therapeutic follow-up both before and after transplantation, whereas others have relied on existing local support networks or have not reported a subjective need for ongoing professional support. Peer support has been experienced as valuable, offering both practical guidance for everyday challenges and insight into the transplantation process and recovery.

### 4.5 Emotional responses during rehabilitation

During periods of rehabilitation plateau or medical setbacks, recipients have described transient emotional reactions characterized by frustration, sometimes expressed using the Finnish colloquial term “*vitutus”.* While not directly corresponding to clinical constructs such as depression or anxiety, this expression captures a situational and often short-lived emotional response to adversity rather than sustained psychopathology.

## 5 Reflections on recipients’ perspectives and feedback

### 5.1 Practical challenges in a centralized care model

VCA-specific follow-up is centralized in Finland, although none of the recipients live in the capital. For some, travel distances are substantial. Although routine transplant follow-up is conducted at HUS, recipients depend on local healthcare for general medical needs. Limited familiarity with VCA among regional providers can result in referrals back to the transplant center even for minor issues, adding logistical strain. These challenges highlight the need for stronger collaboration between specialized and local services, including targeted education and clear consultation pathways. Recipient feedback emphasizes the value of accessible, low-threshold communication with the transplant team during the waiting and postoperative periods. One recipient specifically noted the importance of clear written guidance on early signs of rejection, medication side effects, and self-care strategies.

### 5.2 Psychological burden and adaptation

Long waiting periods, shaped by logistical constraints and donor availability, were described as psychologically demanding, with some individuals experiencing a sense of life being “on hold.” Despite these challenges, all recipients reported meaningful subjective improvement following transplantation.

As functional recovery progresses, emerging sensory and motor experiences appear to support the development of a sense of ownership over the graft. At the same time, recipients emphasized that transplantation does not define them entirely. Two recipients explicitly expressed a wish not to be perceived solely through the transplant—as a face or a hand—but as whole individuals with identities, relationships, and life contexts extending beyond the graft.

## 6 Psychosocial domains in VCA candidate assessment

Based on our clinical experience and existing literature on VCA psychosocial assessment, we propose three interrelated domains that are particularly relevant to clinical assessment: psychosocial functioning, cognitive capacity, and social context (see Table 1) (1–4, 6, 7). This framework is our clinical synthesis rather than a proposed standardized classification; it is broadly consistent with international recommendations emphasizing psychological status, patient expectations, informed decision-making, and social support in face transplantation (7).

**Table 1:** A proposal of psychosocial domains relevant to VCA candidate assessment.

Domain	Conceptual definition	Primary assessment targets	Assessment means and methods	Clinical implications	Typical questions
Psychosocial functioning	Psychosocial functioning refers to the emotional, behavioral, interpersonal, and motivational aspects of a person's life. It captures how an individual manages psychological demands, relates to others, and navigates the social and emotional challenges associated with transplantation.	Current and past psychiatric disorders Historical coping strategies Personality traits, emotional regulation, impulse control Body image Capacity for self-reflection/mentalization Assertiveness Previous adherence to medical treatment Expectations Motivation	Assessment by the surgical team Psychiatric assessment Psychological assessment Possible assessment methods include: SCID-I and II interviews; MINI; IES; TAS-20; RS; BAS-2; COPE Inventory; DERS, MZQ + additional psychological tests as needed (e.g., PAI, Wartegg)	Informs suitability for transplantation and guides planning of perioperative psychological support. Facilitates adaptive engagement with the transplant process and long-term adherence.	How has the candidate managed psychological stressors in the past? Are there psychiatric conditions that may affect postoperative adjustment? Does the candidate demonstrate emotional regulation and impulse control? Are expectations realistic and motivations stable? Is there evidence of sustained treatment adherence? Can the candidate reflect on their emotions, motivations, and potential challenges?
Cognitive capacity	Refers to the ability to understand, retain, and apply complex information related to the transplant process.	Comprehension of surgical and medical risks Understanding of long-term immunosuppression Awareness of rehabilitation requirements Neuropsychological functioning: general cognitive functioning (e.g., abstract reasoning, verbal comprehension), executive function/ attention (e.g., cognitive flexibility, problem solving, inhibition control), social cognition (e.g., Theory of Mind), memory, visuospatial skills	Assessment by the surgical team Psychiatric assessment Psychological assessment Neuropsychological assessment	Ensures meaningful participation in informed consent and supports adherence to complex postoperative regimens. Identifies cognitive vulnerabilities that may require tailored support.	Can the candidate understand and weigh the risks and benefits of VCA? Can they retain and apply complex medical information? Do cognitive abilities support informed consent? Are there cognitive vulnerabilities that may compromise postoperative safety or adherence? Is additional support needed for medication management or rehabilitation?
Social context	Encompasses the availability and quality of social support, practical resources for recovery, and the broader societal environment.	Family and social network support Practical resources for recovery (housing, employment or educational status, and financial situation) Peer support Collaboration between specialized and local healthcare Societal attitudes toward VCA	Unstructured interviews by the surgical team, psychologist and psychiatrist Following the media coverage	Social support is crucial for adherence to rehabilitation and immunosuppressive therapy, and for mitigating potential psychological stressors associated with visible grafts.	Does the candidate have reliable social and practical support for recovery? Are housing, finances, and daily structure sufficient for postoperative demands? Is peer or community support available? Can local healthcare collaborate effectively with the transplant center? Are societal or media pressures likely to affect psychological adjustment?

SCID-I and SCID-II, structured clinical interview I and II for DSM disorders; MINI, mini international neuropsychiatric interview; IES, impact of event scale; TAS-20, Toronto alexithymia scale; RS, resilience scale; BAS-2, body appreciation scale 2; COPE Inventory, coping orientation to problems experienced inventory; DERS, difficulties in emotion regulation scale; MZQ, mentalization questionnaire; PAI, personality assessment inventory; Wartegg, Wartegg drawing completion test.

## 7 Discussion

Long-term VCA outcomes depend not only on surgical expertise and immunological management but also on psychosocial factors that influence engagement in treatment, adaptation to a visible graft, and adherence to lifelong follow-up. Our observations suggest that psychosocial suitability should be assessed over time, with attention to sustained motivation, realistic expectations, the capacity to manage the waiting period and postoperative challenges, social support, and accessible communication with the care team. A history of psychiatric disorders, or the presence of mild or manageable symptoms at the time of evaluation, does not appear to hinder successful engagement with the VCA process.

Our observations are broadly consistent with recent international consensus recommendations framing face transplantation as a multidisciplinary, longitudinal process beyond candidate selection and surgery alone (7). The Finnish centralized national structure may promote continuity and consistent decision-making. Early identification of physical and psychosocial challenges is particularly important in VCA given the prolonged demands of rehabilitation, identity adaptation, and adherence. Thus, close coordination between specialized transplant teams and local healthcare providers is essential.

Our observations may contribute to ongoing efforts to develop more standardized approaches to candidate evaluation and follow-up in VCA. We propose that mentalization may provide a useful conceptual framework, although its clinical relevance in VCA remains unexplored. It may help characterize a candidate's capacity to envision life after transplantation and reflect on potential outcomes, benefits, risks, and challenges. The clinical team's adoption of a mentalizing stance, including consideration of how the candidate understands, anticipates, and emotionally processes the procedure, may also support the informed consent process.

While the transplanted face or hand inevitably attracts medical and public attention, recipients reported that transplantation affects their entire life situation rather than only a specific anatomical structure. This perspective underscores the importance of holistic care approaches. Future efforts to harmonize psychosocial assessment in VCA may benefit from combining longitudinal clinical evaluation with standardized outcome measures addressing quality of life, identity and body image, social reintegration, and return to work (7).

Several limitations should be acknowledged. This perspective is based on clinical observations and professional reflection within a small national program involving highly selected candidates and recipients, which limits its generalizability. Candidates screened out by the surgical team were not encountered in our clinical practice and fall outside the scope of the article. Formal psychometric evaluations were not conducted systematically in the follow-up period. Patient-reported outcomes and feedback were collected through routine clinical encounters rather than using standardized research methods.

VCA raises important ethical questions because it is life-enhancing rather than life-saving and entails lifelong immunosuppression and potentially life-threatening complications (19). A detailed ethical analysis was, however, beyond the scope of this article.

## 8 Conclusions

Taken together, the Finnish experience supports a process-oriented model of psychosocial evaluation in VCA. We propose that the assessment address three interrelated domains: psychosocial functioning, cognitive capacity, and social context. We also propose mentalization as a potentially useful conceptual perspective. Psychosocial evaluation should inform candidate selection and preparation, as well as the ongoing support and reassessment of candidates and recipients throughout the transplantation process. Limited psychiatric morbidity and variation in support needs further suggest that psychosocial care should be individualized.

## Data Availability

The original contributions presented in the study are included in the article/Supplementary Material, further inquiries can be directed to the corresponding author.
